# Examining the influence of family physician supply on district health system performance in South Africa: An ecological analysis of key health indicators

**DOI:** 10.4102/phcfm.v9i1.1298

**Published:** 2017-04-28

**Authors:** Klaus B. von Pressentin, Robert J. Mash, Tonya M. Esterhuizen

**Affiliations:** 1 Division of Family Medicine and Primary Care, Stellenbosch University, South Africa; 2 Biostatistics Unit, Faculty of Medicine and Health Sciences, Stellenbosch University, South Africa

## Abstract

**Background:**

The supply of appropriate health workers is a key building block in the World Health Organization’s model of effective health systems. Primary care teams are stronger if they contain doctors with postgraduate training in family medicine. The contribution of such family physicians to the performance of primary care systems has not been evaluated in the African context. Family physicians with postgraduate training entered the South African district health system (DHS) from 2011.

**Aim:**

This study aimed to evaluate the impact of family physicians within the DHS of South Africa. The objectives were to evaluate the impact of an increase in family physician supply in each district (number per 10 000 population) on key health indicators.

**Setting:**

All 52 South African health districts were included as units of analysis.

**Methods:**

An ecological study evaluated the correlations between the supply of family physicians and routinely collected data on district performance for two time periods: 2010/2011 and 2014/2015.

**Results:**

Five years after the introduction of the new generation of family physicians, this study showed no demonstrable correlation between family physician supply and improved health indicators from the macro-perspective of the district.

**Conclusion:**

The lack of a measurable impact at the level of the district is most likely because of the very low supply of family physicians in the public sector. Studies which evaluate impact closer to the family physician’s circle of control may be better positioned to demonstrate a measurable impact in the short term.

## Introduction

Strong primary health care systems require primary care teams that consist of an appropriate mix of health workers tailored to the health care needs of the communities they work in.^1^ The supply of appropriate health workers is a key building block in the World Health Organization’s (WHO) model of effective health systems.^2^ In sub-Saharan African countries these primary care teams and their communities are challenged by a mix of health system constraints, socio-economic disparities and disease burdens.^3^ Primary care teams are stronger if they contain doctors with postgraduate training in family medicine.^2^ The contribution of such family physicians to the performance of primary care systems has been established in high-income countries.^4,5^ International studies (mainly in the USA, UK, Canada and Korea) described the public health benefits associated with an increased supply of primary care doctors, especially regarding a reduction in all-cause, infant and chronic disease-related morbidity and mortality.^6,7,8,9,10,11,12,13^ Many of these studies applied a broad definition of primary care doctors, by including all clinical specialities that work in primary care (family medicine, general practice, general internal medicine and general paediatrics).^6,7,10,12,14^ Some of these studies (notably UK and Canada) focused on family physicians or general practitioners, two terms which apply to the same professional: a primary care doctor with postgraduate training in family medicine or general practice.^9,11,13^

Family medicine is a young discipline in Africa, with a number of countries only commencing postgraduate training during the last decade.^3,15,16,17,18,19,20,21,22^ Qualitative studies have explored the opinions of African leaders and managers on the potential contribution and possible roles of family physicians in the district health system (DHS).^23,24,25,26^ There is, however, little quantitative evaluation of their actual impact to guide policy- and decision-makers on the deployment of family physicians. The uncertainty revolves around their cost-effectiveness and how best to position these family physicians within the different levels and components of the health system. The relationship between family physician supply and DHS performance has not been evaluated in the African context.

In South Africa, family medicine was gazetted as a new speciality during 2007 by the Health Professions Council of South Africa (HPCSA).^5,19^ This event paved the way for structured postgraduate training through training posts (registrars) and a consensus on training outcomes.^5^ This developmental phase included the creation of new family physician posts within the DHS. These posts are mainly at district hospitals and community health centres, although a few are located at regional hospitals. During this same period, the National Department of Health (NDoH) started implementing primary health care reforms, which included family physicians within district clinical specialist teams that were tasked with strengthening maternal and child health care.^27,28,29^ In addition, the new national policy on human resources for health and the national development plan support the deployment of family physicians within the DHS, but lack sufficient detail to guide managers on how best to utilise these expert generalists.^30^ Following further discussions with the national department, a national position paper was published by the leadership of academic family medicine, in order to clarify the contribution of family physicians to the DHS.^5^ This consensus statement introduced the ‘new’ definition of the family physician as an expert generalist in the DHS capable of supporting and leading health care teams through six interwoven roles: competent clinician, consultant to the primary care team, capacity builder, leader of clinical governance, supporter of community-orientated primary care and in some instances a supervisor of under- or postgraduate students.

The first graduates of the new training programmes entered the DHS from 2011.^5^ Family physicians from the previous training programmes in South Africa and elsewhere still form the bulk of the available family physicians, as the nine South African training institutions are not yet training to the scale envisaged by the national position paper.^5^ The training standards are coordinated through the South African Academy of Family Physicians (SAAFP), and the South African College of Family Physicians (CFP) is responsible for the national exit examination. The nine training institutions, SAAFP and CFP successfully responded to a funding call from the NDoH and EuropeAid to implement a project aimed at strengthening the contribution of family physicians to the primary health care system.^31^ This project included an applied research activity, which aimed to evaluate the initial impact of family physicians on the DHS in South Africa. This article presents one of the four complementary studies and looks at the relationship between the supply of family physicians and DHS performance. The other three studies consist of a quasi-experimental comparison of facilities with and without family physicians, a 360-degree evaluation of family physician’s impact by their colleagues and qualitative interviews with district managers who employ family physicians.

### Aim and objectives

This study aimed to evaluate the impact of family physicians within the DHS of South Africa. The objectives were to evaluate the impact of an increase in family physician supply in each district (number per 10 000 population) on key health system performance indicators, key clinical processes and key health outcomes.

## Research methods and design

### Study design

This ecological study was informed by a pilot study conducted in the Western Cape, South Africa.^32^ A retrospective cohort design was used, whereby data were collected for the period 2010/2011 as a baseline and 2014/2015 representing 5 years post-deployment of the new generation of family physicians. The STROBE statement’s checklist for reporting cohort studies was used as standard for presenting this research.^33^

### Setting

**FIGURE 1:**
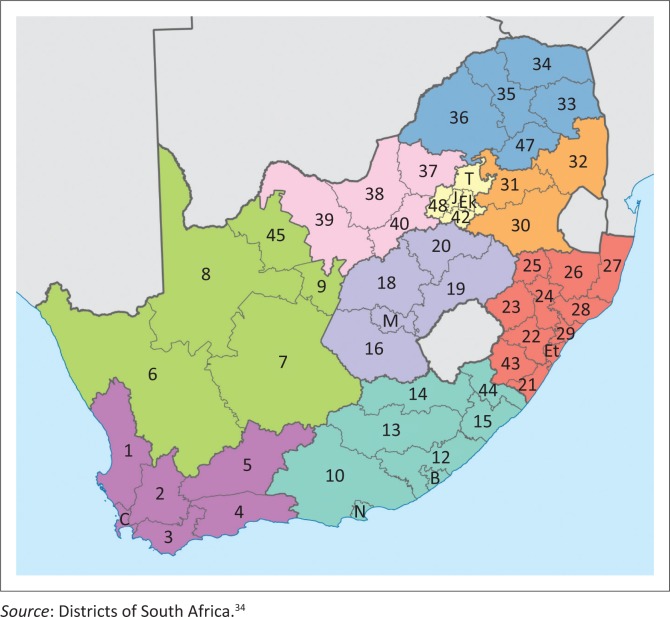
Map of South Africa depicting its 52 districts.

This study evaluated all 52 health districts across all nine provinces of South Africa (a national study frame, see Figure 1) for two time periods.

### Study population and sampling strategy

All 52 South African health districts were included as units of analysis.

### Data collection

A national dataset, the District Health Barometer (DHB), contributed the data on district performance for two time periods: 2010/2011 and 2014/2015.^35,36^ The DHB draws data from several data sources provided by the NDoH. Compilation of the DHB is guided by an advisory committee made up of managers from the NDoH, as well as health experts from Health Systems Trust (HST). The DHB is designed to assist the NDoH in monitoring health service delivery at district level for all of South Africa’s health districts. Furthermore, the HST encourages providers, managers, researchers and policy-makers to use DHB information by making the publication and its data freely available online on their website.

Table 1 presents the list of DHB indicators used. The DHB system of categorising the indicators was used throughout (ranging from financial indicators to clinical process and outcome indicators). The official DHB indicator descriptions are also presented in Table 1.

**TABLE 1:** List of DHB data indicators arranged by DHB categories.^35^

Category	DHB indicator name	DHB 2014/2015 description of the indicators
Finance	Provincial and LG PHC expenditure per PHC headcount^a^	Provincial and LG expenditure under programme 2 (budget for District Health Services) per PHC headcount on non-hospital PHC divided by the total PHC headcount. PHC programmes include nutrition; HIV and AIDS; community-based services; community health centres; and community health clinics.
	Provincial and LG expenditure on District Health Services per capita (uninsured)^a^	Provincial and LG expenditure per capita (uninsured) on DHS is the total amount spent per person without medical aid coverage. The numerator is the sum of provincial and LG expenditure under programme 2, except for expenditure on sub-programme 2.8 (Coroner Services). The denominator is the estimated uninsured population per district. Uninsured individuals have no medical scheme coverage.
	Provincial and LG PHC expenditure per capita (uninsured)^a^	PHC expenditure for the uninsured population includes expenditure on sub-programmes 2.2–2.7 of the DHS expenditure. This forms the numerator for this indicator. The denominator is the estimated uninsured population per area.
	Provincial and LG expenditure on District Health Services per capita (total population)^a^	The provincial and LG district expenditure on DHS per capita (total population) refers to the total amount of money spent on DHS (all sub-programmes except 2.8 Coroner services) per person with and without medical scheme coverage.
	Provincial and LG PHC expenditure per capita (total population)^a^	The PHC expenditure per capita (total population) measures the total amount of money spent annually by each district as a percentage of the total population in the district.
Management PHC	PHC supervisor visit rate (fixed clinic/CHC/CDC)^a^	The PHC facility supervision rate is the number of fixed PHC facilities, including CHCs and CDCs, visited by a clinical supervisor at least once a month, as a proportion of the total number of fixed PHC facilities. A dedicated clinic supervisor conducts the visit according to the clinic supervision manual, which entails use of the red flag and/or regular review tools. Each fixed facility should be visited by a clinic supervisor once a month.
Management Inpatients	ALOS (district hospitals)^a^	ALOS refers to the average number of days that patients spend in hospital. It is generally calculated as follows: total number of inpatient days during a year plus half the number of day patients, divided by the number of separations (deaths, discharges and transfers out).
	Inpatient bed utilisation rate (district hospitals)^a^	BUR measures the occupancy of available beds and therefore indicates how efficiently a hospital is using its available capacity. It is calculated as follows: the number of inpatient days is added to half the number of day patients, and divided by the usable bed days; this is expressed as a percentage.
	OPD new client not referred rate (district hospitals)^a^	OPD new client not referred rate refers to the percentage of new outpatient clients who enter a hospital without a referral letter. The percentage is calculated by dividing new OPD cases that are not referred (numerator) by all new OPD cases (denominator). OPD follow-up and emergency clients are excluded from the denominator. OPD new client not referred rate monitors the utilisation trends of clients who by-pass PHC facilities.
	Expenditure per PDE (district hospitals)^a^	Expenditure per PDE is a composite process indicator that connects financial data with service-related data from the hospital admissions and outpatients’ records. This indicator measures how the resources available to the hospital are being spent and is a marker of efficiency. The indicator measures the average cost per PDE at a district hospital and is expressed as Rand per PDE. The indicator value is calculated by dividing the total expenditure of the hospital (within budget programme 2: district health services, as recorded in the BAS) by the number of PDEs. PDEs are calculated by adding the number of inpatients, plus half of day patients, plus one-third of outpatients and emergency room visits, as recorded in the DHIS. As expenditure per PDE is a ratio between costs and services, improved performance is possible if costs are reduced or utilisation increased.
Inpatient mortality	Child under 5 years diarrhoea case fatality rate^a^ Child under 5 years pneumonia case fatality rate^a^ Child under 5 years severe acute malnutrition case fatality rate^a^	CFRs for diarrhoea, pneumonia and SAM in children under 5 years of age. The CFR for the priority childhood illnesses (pneumonia, diarrhoea and SAM) is the proportion of all children under 5 years admitted to hospital with these conditions that die during the admission.
	ICDR	The ICDR is an impact indicator that refers to the proportion of all inpatient separations because of death. Inpatient separations include inpatient transfers out, deaths and inpatient discharges. The indicator therefore includes deaths from all causes that occur in a health facility.
Delivery care	Delivery in facility under 18 years rate^a^	This indicator measures the proportion of all deliveries that occur among women younger than 18 years. The numerator is the number of deliveries among women under 18 years of age, while the denominator represents all deliveries that have been recorded at the health facility. This outcome indicator is used as a proxy to track success in the prevention of teenage pregnancies.
	Inpatient ENDR^a^	The inpatient ENDR or inpatient death 0–7 days measures the number of deaths among live born babies that occur within seven completed days after birth per 1000 live births. It only includes neonatal deaths when the foetus is at 26 or more weeks’ gestational age and/or weighs 500 g or more.
	Maternal mortality in facility ratio^a^	The WHO definition of a maternal death is the death of a woman while pregnant or within 42 days of termination of pregnancy, irrespective of the duration and site of the pregnancy, from any cause related to or aggravated by the pregnancy or its management, but not from accidental or incidental causes. The MMR is the number of maternal deaths per 100 000 live births. This indicator refers to the facility-based (and not the population-based) MMR.
	Stillbirth in facility rate^a^	The stillbirth rate measures the number of babies born dead per 1000 total births. The indicator does not differentiate between fresh and macerated stillbirths. Stillbirths should only be counted when the foetus is at 26 or more weeks of gestational age and/or weighs 500 g or more.
	Delivery by C-section rate (district hospitals)^a^	The C-section rate measures the proportion of deliveries in hospitals that are carried out by C-section. The numerator is the number of C-sections conducted in the facility, and the denominator is the number of deliveries that took place in that facility over the same time period. It is therefore a facility-based and not a population-based indicator. This chapter focuses on C-sections performed at district hospitals.
	Mother postnatal visit within 6 days rate^a^	The mother postnatal visit within 6 days rate indicator monitors access to postnatal care. The numerator for this indicator is the number of postnatal visits by a mother within 6 days of delivery, either at a PHC facility or a postnatal home visit by facility staff. The purpose of the visit is for a postnatal check-up. Only the first visit after delivery should be counted. The denominator is the number of deliveries in facility. Deliveries include deliveries at hospitals and at PHC facilities.
PMTCT	Antenatal first visit before 20 weeks rate^a^	Early registration for antenatal care is an important entry point into the health system for pregnant women, allowing them to access health care services (and health information), including PMTCT services. This indicator shows the percentage of pregnant women who have their first antenatal visit before 20 weeks, out of all antenatal clients’ first visits (those whose first visit was before and after 20 weeks).
	Antenatal client initiated on ART rate^a^	All HIV-positive pregnant women should be initiated on ART at the first antenatal visit if not already on ART. The antenatal client initiated on ART rate indicator measures the percentage of antenatal clients initiated on ART out of all antenatal clients eligible for ART.
	Infant first PCR test positive around 6 weeks rate^a^	This indicator measures the percentage of HIV-exposed infants who receive an early HIV test (around 6 weeks of age). It is calculated by dividing the number of PCR tests performed in infants around 6 weeks (numerator) by live births to HIV-positive women (denominator). It can be used as a proxy for early infant diagnosis coverage.
	Infant first PCR test around 6 weeks uptake rate^a^	This indicator measures the percentage of early infant PCR tests that have a positive result; it is used as a proxy for early vertical (intra-uterine and intra-partum) transmission for those infants who access an early PCR test.
Child Health	Vitamin A dose 12–59 months coverage (annualised)^a^	Proportion of children 12–59 months who received vitamin A 200 000 units, preferably every 6 months.
	School Grade 1 screening coverage (annualised)	Proportion of Grade 1 learners screened by a nurse in line with the Integrated School Health Programme service package.
Immunisation	Immunisation coverage under 1 year^a^ Measles second dose coverage (annualised)^a^	Immunisation coverage under 1 year measures the percentage of children under 1 year old who have received the primary schedule of immunisations.
Reproductive health	Cervical cancer screening coverage (annualised)^a^	The cervical cancer screening coverage measures the annual number of cervical smears taken in women 30 years and older as a proportion of the female population 30 years and older, factored for one smear every 10 years. In practice this means that the denominator is 10% of the female population aged 30 years and older.
	CYPR (annualised)^a^	The CYPR indicator measures the percentage of women aged from 15 to 49 years who are protected against unplanned pregnancies for a year using modern contraceptive methods, including sterilisation. The volume of all contraceptives dispensed to clients during a specified period of time (a year) is used to estimate the amount of protection against pregnancy during that particular period. This estimate of protection is called the ‘contraceptive year equivalent’. This forms the numerator for the CYPR indicator. Each type of contraceptive method that is distributed is adjusted by a conversion factor (country-specific) to yield an estimate of the duration of contraceptive protection. The denominator for the CYPR is the ‘female target population 15–49 years’, where females are used as a proxy for couples.
Tuberculosis case finding	Incidence (diagnosed cases) of TB – all types^a^	The number of TB patients (all TB types) starting treatment and recorded in the Electronic TB Register (ETR.Net).
	TB Rifampicin resistance confirmed client rate	This indicator measures the proportion of TB suspects detected to have rifampicin resistance. In 2011, GeneXpert diagnostic machines were introduced across South Africa; these machines can detect both TB and rifampicin resistance in just 2 hours. The rifampicin resistance confirmed client rate was reported for the first time in the 2013/14 DHB.
HIV management	Male condom distribution coverage^a^	Male condom distribution coverage refers to the number of male condoms distributed through public health facilities, identified outlets and other non-medical sites in a given 12-month period per male aged 15 years and older. Distribution of condoms remains an integral and cost-effective component of South Africa’s HIV prevention efforts.
	Percentage of TB cases with known HIV status (ETR.net)^a^	This indicator measures the percentage of TB cases with known HIV status entered into the ETR.Net system.
	TB/HIV co-infected client on ART rate (ETR.Net)	The TB/HIV co-infected client on ART indicator entered into the ETR.Net system measures the percentage of all HIV-positive TB patients on ART. It is an important indicator that may be used as a proxy for measuring integration of HIV and TB services.
	HIV testing coverage (including ANC)	The HIV testing coverage indicator measures all people aged from 15 to 49 years who were tested for HIV (including antenatal care) during the year as a percentage of the total population in this age group. People are tested either through provider-initiated or client-initiated counselling and testing services.
Non-communicable diseases	Hypertension incidence (annualised)	This indicator measures the number of newly diagnosed hypertension clients initiated on treatment per 1000 population 40 years and older. The numerator is ‘hypertension client treatment new’ and the denominator is ‘population 40 years and older’.
	Mental health admission rate	The mental health admission rate indicator measures the proportion of clients admitted/separated for mental health problems. The numerator is the ‘mental health admissions total’ and the denominator is ‘inpatient separations total’ (total of inpatient discharges, inpatient deaths and inpatient transfer outs).
Human resources	PHC doctor clinical work load	The PHC doctor clinical workload is expressed as the number of consultations (clients) per doctor per day.
	PHC PN clinical work load	PN clinical workload is defined as the average number of clients attended by all PNs in a PHC facility per day. The numerator for this indicator is expressed as the total number of clients seen at a PHC facility, while the denominator is the total number of PN clinical work days. This is a useful indicator to measure the efficiency of PHC services rendered to clients, and to analyse PHC utilisation patterns, staffing and training needs.
Additional indicators reported in the DHB 2014/2015 dataset	PCV third dose coverage (annualised)^a^	PCV vaccine third dose given to a child under 1 year, preferably around 9 months after birth.
	Percentage of DHS expenditure on district hospitals^a^	Percentage of total provincial district health services expenditure on district hospitals.
	Percentage of DHS expenditure on district management^a^	Percentage of total provincial district health services expenditure on district management.
	Percentage of DHS expenditure on PHC^a^	Total amount spent on non-hospital PHC health services.
	RV second dose coverage (annualised)^a^	RV vaccine second dose given to a child under 1 year, preferably around 14 weeks after birth and not later than 24 weeks after birth.
	HIV prevalence among antenatal clients (survey)	Proportion of antenatal clients surveyed who test positive for HIV.
	Vaccine expenditure per population under 1 year^a^	Expenditure (in Rand) per child fully immunised under 1 year of age (immunised according to the routine Expanded Programme on Immunisation).
	HIV testing coverage (annualised)	Clients HIV tested as proportion of population 15–49 years.
	Tracer items stock-out rate (fixed clinic/CHC/CDC)	The availability of a trace list of essential medicines (this measure of medicine shortages is routinely reported).
	TB/HIV co-infected client on ART mm rate	Proportion of TB/HIV co-infected clients initiated on ART.

*Source:* The definitions of the indicators were adopted from Massyn^35^

a Indicators available for both time periods.

ALOS, average length of stay; ANC, antenatal care; ART, antiretroviral therapy; BAS, Basic Accounting System; BUR, bed utilisation rate; CFRs, case fatality rates; CHC, community health centre; CDC, community day centre; C-section, caesarean section; CYPR, couple year protection rate; DHIS, District Health Information Software; DHB, District Health Barometer; DHS, District Health System; ENDR, early neonatal death rate; ETR.Net, Electronic TB Register; ICDR, inpatient crude death rate; LG, local government; MMR, maternal mortality ratio; OPD, outpatient department; PCR, polymerase chain reaction; PCV, pneumococcal vaccine; PDE, patient day equivalent; PHC, primary health care; PMTCT, prevention of mother-to-child transmission; PN, professional nurse; RV, Rota virus; SAM, severe acute malnutrition; TB, tuberculosis; WHO, World Health Organization.

For the family physician supply, public sector family physicians working in joint appointments (with the universities) or non-joint appointments and employed at facility-, sub-district and district levels (including district office and district clinical specialist team appointments) were included. Those family physicians employed at regional or tertiary hospitals in full-time academic positions or in the private sector were excluded. The data on family physician supply per district for these two time periods were obtained from all nine academic institutions involved with postgraduate family medicine training in South Africa and who were familiar with the health system in their catchment area. The absolute numbers of family physicians were converted to family physician supply per 10 000 population (using the DHB population data for the respective time periods).

### Data analysis

The DHB data, as well as data on family physician supply, were entered into an Excel sheet and subsequently converted into IBM SPSS version 23 for descriptive and inferential analyses.^37^

The data analysis included all 52 units of analysis and commenced with descriptive analysis of the independent and dependent variables. Subsequently, the correlation between change in family physician supply and change in the indicators available for both time periods (37 indicators) was analysed. In addition, a cross-sectional correlation analysis was performed for time period 2 (2014/2015) on the remaining DHB data set (data for 12 indicators were available only for time period 2). Simple scatterplots of the bivariate correlations were inspected to identify the nature of each relationship. A non-parametric test, Spearman’s rho, was selected to test for correlation between the independent and dependent variables, because of the non-parametric distribution of the data as well as the presence of outliers (especially in reference to the independent variable). The level of significance chosen was *p* < 0.05. For those relationships found to be linear and showing at least a low-to-moderate correlation coefficient (see interpretation guide below), further regression analysis was performed using a generalised linear model (GLM), to control for the effect of available confounders, namely province and socio-economic quintile (SEQ) of the districts. Using GLMs with province as covariate created better regression models as opposed to GLMs with SEQ as covariate (using the omnibus test and its likelihood ratio Chi-square value as guide).

Correlation values may be interpreted as:^32,38^

**Table:** 

0.90–1.00 (−0.9 to −1.00)	Very high positive (negative) correlation
0.70–0.90 (−0.70 to −0.90)	High positive (negative) correlation
0.50–0.70 (−0.50 to −0.70)	Moderate positive (negative) correlation
0.30–0.50 (−0.30 to −0.50)	Low positive (negative) correlation
0.00–0.30 (0.00 to −0.30)	Negligible correlation

### Ethical considerations

This study was approved by the Health Research Ethics Committee, Stellenbosch University (reference S15/01/003) and HST also confirmed their permission for use of the open access data.

## Results

**FIGURE 2:**
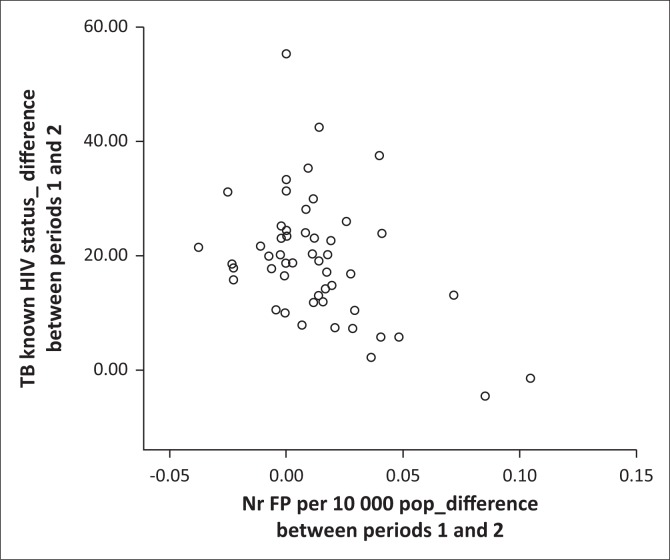
Scatter plot of significant correlation (*p* < 0.05): difference between time periods 1 and 2 for supply of family physicians (FPs) and percentage of TB cases with known HIV status.

**FIGURE 3:**
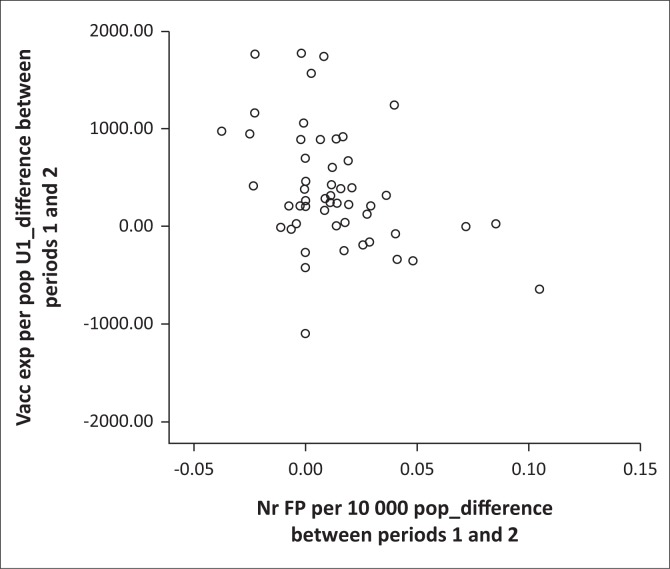
Scatter plot of significant correlation (*p* < 0.05): difference between time periods 1 and 2 for supply of family physicians (FPs) and vaccine expenditure per population under 1 year.

**FIGURE 4:**
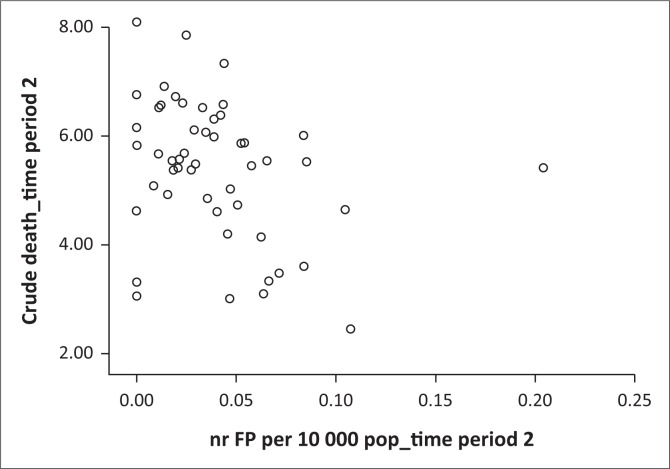
Scatter plot of significant correlation (*p* < 0.05): supply of family physician (FPs) and inpatient crude death rate for time period 2 (2014/2015).

Tables 2 and 3 present descriptive statistics for the dependent variables, as well as the results for the non-parametric correlation analysis. The median (and interquartile range) of the independent variable, the supply of family physician per 10 000 total population, was 0.027 (0.000–0.043) for time period 1 and 0.035 (0.016–0.054) for time period 2. The medians (and interquartile ranges) for the absolute numbers of family physicians per district were 2.00 (0.00–4.00) for time period 1 and 2.00 (1.00–5.00) for time 2 (total numbers were 153.5 for time period 1 and 208.5 for time period 2). The majority of correlations were negligible to low and not statistically significant. Two correlations from the change over time correlation analysis were found to be statistically significant (using the initial Spearman’s rho analysis): a HIV management indicator, ‘Percentage of TB cases with known HIV status’ (low negative correlation, rho = -0.351, *p* = 0.011) and an additional indicator, ‘Vaccine expenditure per population under 1 year’, a measure of the efficiency of immunisation and not the coverage (low negative correlation, rho = -0.378, *p* = 0.006). One indicator from the cross-sectional time 2 analysis showed a statistically significant, low negative correlation, namely ‘Inpatient crude death rate’ (rho = -0.340, *p* = 0.014). Scatter plots of these correlations are shown in Figures 2, Figure 3 and Figure 4. The influence of the three outlying values was clear on inspection: for example, the scatterplot of ‘Percentage of TB cases with known HIV status’ (Figure 2) showed a random scatter if one ignores the three outliers.

**TABLE 2:** Correlations: difference over time (37 variables available for both time periods).

DHB indicator name (unit)	2010/2011 Median (IQR)	2014/2015 Median (IQR)	Spearman’s rho	*p*
Financial indicators				
Provincial and LG PHC expenditure per PHC headcount (Rand)	262.78 (232.49–291.32)	314.15 (276.35–342.80)	0.192	0.174
Provincial and LG expenditure on District Health Services per capita (uninsured) (Rand)	1430.15 (1232.31–1571.91)	1600.22 (1351.84–1895.19)	0.015	0.917
Provincial and LG PHC expenditure per capita (uninsured) (Rand)	761.89 (672.41–828.93)	929.56 (794.46–1018.46)	0.136	0.336
Provincial and LG expenditure on District Health Services per capita (total pop) (Rand)	1218.82 (1028.83–1462.29)	1341.33 (1149.76–1737.68)	0.012	0.933
Provincial and LG PHC expenditure per capita (total pop) (Rand)	629.89 (577.72–713.14)	755.34 (674.76–898.70)	0.132	0.351
Management of PHC				
PHC supervisor visit rate (fixed clinic/CHC/CDC) (%)	66.50 (54.09–83.73)	77.53 (62.15–85.22)	0.125	0.376
Management of inpatients				
Average length of stay (district hospitals) (days)	4.02 (3.04–5.22)	4.32 (3.51–5.37)	−0.205	0.145
Inpatient bed utilisation rate (district hospitals) (%)	64.42 (60.57–71.62)	66.70 (59.33–73.18)	−0.83	0.557
OPD new client not referred rate (district hospitals) (%)	63.98 (35.39–82.41)	59.87 (42.94–70.15)	−0.148	0.337
Expenditure per patient day equivalent (district hospitals) (Rand)	1925.71 (1706.56–2163.24)	2078.39 (1918.54–2420.58)	0.052	0.715
Inpatient mortality				
Child under 5 years diarrhoea case fatality rate (%)	7.81 (3.19–10.05)	2.97 (1.79–4.68)	0.73	0.608
Child under 5 years pneumonia case fatality rate (%)	6.22 (3.09–9.03)	2.66 (1.61–4.45)	−0.085	0.548
Child under 5 years severe acute malnutrition case fatality rate (%)	17.46 (10.76–23.12)	11.14 (8.27–15.04)	0.005	0.975
Delivery care				
Delivery in facility under 18 years rate (%)	8.58 (7.25–10.22)	8.00 (6.97–9.81)	0.098	0.49
Inpatient early neonatal death rate (per 1000 live births)	9.64 (8.26–13.04)	10.27 (8.34–12.40)	0.14	0.321
Maternal mortality in facility ratio (per 100 000 live births)	132.51 (58.38–197.22)	130.21 (69.80–195.03)	0.036	0.802
Stillbirth in facility rate (%)	22.95 (18.87–25.98)	20.88 (16.99–24.15)	−0.143	0.312
Delivery by caesarean section rate (district hospitals) (%)	18.43 (13.13–22.20)	21.86 (18.94–27.33)	−0.19	0.177
Mother postnatal visit within 6 days rate (%)	29.28 (11.84–44.09)	69.31 (56.48–76.00)	0.056	0.695
PMTCT				
Antenatal first visit before 20 weeks rate (%)	40.38 (34.98–45.55)	56.95 (52.54–60.89)	−0.148	0.295
Antenatal client initiated on ART rate (%)	74.63 (52.08–109.10)	92.21 (87.41–95.98)	0.052	0.716
Infant first PCR test positive around 6 weeks rate (%)	5.61 (4.98–8.26)	1.54 (1.32–1.95)	0.026	0.855
Infant first PCR test around 6 weeks uptake rate (%)	89.15 (76.92–99.44)	97.89 (89.97–107.98)	0.038	0.791
Child health immunisation				
Vitamin A dose 12–59 months coverage (annualised) (proportion of children aged 12–59 months)	32.98 (26.00–38.14)	51.01 (46.56–58.25)	0.086	0.544
Immunisation coverage under 1 year (%)	77.35 (70.11–88.55)	82.88 (78.77–92.92)	−0.107	0.452
Measles second dose coverage (annualised) (%)	78.96 (72.62–85.94)	79.28 (73.59–87.72)	0.134	0.343
Reproductive health				
Cervical cancer screening coverage (annualised) (proportion of the female population 15–44 years)	49.20 (39.36–61.74)	54.73 (41.68–66.30)	−0.146	0.3
Couple year protection rate (annualised) (proportion of the female population 30 years and older)	28.98 (25.56–36.39)	45.92 (39.61–52.37)	0.026	0.854
TB case finding				
Incidence (diagnosed cases) of TB – all types (per 100 000 people in the catchment population)	919.30 (653.34–1063.57)	680.27 (504.10–831.39)	0.019	0.893
HIV management				
Male condom distribution coverage (number of male condoms)	12.28 (8.93–16.04)	36.78 (24.49–46.53)	0.087	0.542
Percentage of TB cases with known HIV status (ETR.net) (%)	73.11 (68.25–79.52)	93.07 (90.73–94.93)	−0.351	0.011*
Additional indicators				
PCV third dose coverage (annualised) (%)	74.95 (63.06–82.80)	86.09 (81.20–96.20)	0.047	0.74
Percentage of DHS expenditure on district hospitals (%)	44.23 (33.75–49.42)	37.99 (27.58–48.16)	−0.038	0.791
Percentage of DHS expenditure on district management (%)	5.57 (2.90–6.89)	5.49 (3.24–8.06)	0.094	0.507
Percentage of DHS expenditure on PHC (%)	53.88 (45.80–61.08)	58.00 (48.21–66.74)	0.006	0.968
RV second dose coverage (annualised) (%)	72.57 (61.76–82.77)	89.32 (82.89–100.08)	0.072	0.612
Vaccine expenditure per population under 1 year (Rand)	925.74 (0.35–1278.64)	1282.37 (902.57–1445.37)	−0.378	0.006*

* , Statistically significant at *p* < 0.05.

IQR, interquartile range; LG, local government; DHB, District Health Barometer; PMTCT, prevention of mother-to-child transmission; PCR, polymerase chain reaction; DHS, District Health System; PCV, pneumococcal vaccine; RV, Rota virus; ETR.Net, Electronic TB Register; PHC, primary health care; CHC, community health centre; CDC, community day centre; OPD, outpatient department; LG, local government**.**

**TABLE 3:** Cross-sectional correlations time period 2 (12 additional variables only available for time period 2).

DHB indicator name (unit)	2014/2015 Median (IQR)	Spearman’s rho	p
Inpatient mortality			
Inpatient crude death rate (proportion of all inpatient separations)	5.54 (4.66–6.36)	−0.34	0.014*
Child health immunisation			
School Grade 1 screening coverage (annualised) (%)	21.37 (13.31–32.69)	0.23	0.102
TB case finding			
TB rifampicin resistance confirmed client rate (% of positive TB tests that are rifampicin resistant)	5.95 (4.76–7.04)	−0.052	0.712
HIV care			
TB/HIV co-infected client on ART rate (ETR.Net) (%)	81.32 (70.21–86.83)	−0.261	0.061
HIV testing coverage (including ANC) (%)	32.84 (27.04–41.33)	0.012	0.931
NCD care			
Hypertension incidence (annualised) (per 1000 population 40 years and older)	14.82 (11.82–17.69)	−0.18	0.201
Mental health admission rate (proportion of clients admitted/separated for mental health problems)	0.96 (0.53–1.72)	−0.066	0.641
Human resources			
PHC doctor clinical work load (average number of clients seen per doctor per clinical work day)	25.46 (19.05–32.87)	0.073	0.608
PHC professional nurse clinical work load (average number of clients seen per professional nurse per clinical work day)	28.80 (25.40–35.33)	−0.071	0.616
Additional indicators			
HIV testing coverage (annualised) (%)	29.39 (24.39–37.22)	0.036	0.799
Tracer items stock-out rate (fixed clinic/CHC/CDC) (%)	16.35 (8.26–32.69)	−0.131	0.353
TB/HIV co-infected client on ART rate (%)	48.44 (35.37–59.34)	0.221	0.14

* , Statistically significant at *p* < 0.05.

IQR, interquartile range; DHB, District Health Barometer; TB, tuberculosis; ANC, antenatal care; ART, antiretroviral therapy; ETR.Net, Electronic TB Register; PHC, primary health care; CHC, community health centre; CDC, community day centre; NCD, Non-Communicable Disease.

Regression analysis of these three correlations was performed. After adjusting for province in a GLM, the overall vaccine expenditure became positive in most of the nine provinces (see Table 4). This is a real example of confounding by province. Relative to the Western Cape Province, most of the provinces increased their expenditure on vaccines between time periods 1 and 2. The effect of family physicians (not statistically significant at *p* = 0.861) only accounted for an additional R268.249 (after subtracting the intercept value R107.949 from the B coefficient, R376.198). A similar influence of province on the correlation between family physician supply for time period 2 and ‘Inpatient crude death rate’ was demonstrated in a different GLM (Table 5). The correlation remained negative, but decreased in its strength and became non-significant (B coefficient for family physician supply in time period 2 was -0.024 with *p* = 0.334; intercept B coefficient = 3.250). The influence of province on the correlation between family physician supply over time and ‘Percentage of TB cases with known HIV status’, however, was not demonstrated in a GLM (Table 6). Here the B coefficient for change in family physician supply was -138.039% with *p* = 0.029; intercept B coefficient = 15.143%. The overall significance of the provincial covariate was *p* = 0.810 (Wald Chi-Square test).

**TABLE 4:** Generalised linear model (regression analysis) to control for the effect of province on the correlation between changes in family physician supply per 10 000 population and vaccine expenditure per population under 1 year.

Parameter	B	s.e.	95% Wald confidence interval		Hypothesis test		
			Lower	Upper	Wald Chi-square	df	Sig.
(Intercept)	−107.949	104.9363	−313.620	97.722	1.058	1	0.304
FPppop_change	376.198	2153.2942	−3844.181	4596.577	0.031	1	0.861
[Province=EC]	402.050	134.5360	138.365	665.736	8.931	1	0.003
[Province=FS]	9.567	155.9614	−296.111	315.246	0.004	1	0.951
[Province=GP]	1611.691	149.7983	1318.092	1905.290	115.758	1	0.000
[Province=KZN]	424.724	123.3641	182.935	666.513	11.853	1	0.001
[Province=LP]	1023.000	150.5760	727.876	1318.123	46.157	1	0.000
[Province=MP]	155.624	170.7158	−178.973	490.220	0.831	1	0.362
[Province=NC]	−355.870	170.6256	−690.290	−21.450	4.350	1	0.037
[Province=NW]	1210.934	160.8308	895.711	1526.156	56.689	1	0.000
[Province=WC]	0^a^						
(Scale)	57991.602^b^	11716.0729	39029.809	86165.575			

Dependent variable: Vacc exp per pop U1_difference Model: (Intercept), FPppop_change, Province.

a , Set to zero because this parameter is redundant.

b , Maximum likelihood estimate.

Sig., significance level; df, degrees of freedom; s.e., standard error; B, regression; EC, Eastern Cape; FS, Free State; GP, Gauteng Province; KZN, KwaZulu-Natal; LP, Limpopo Province; MP, Mpumalanga; NC, Northern Cape; NW, North West; WC, Western Cape.

**TABLE 5:** Generalised linear model (regression analysis) to control for the effect of province on the correlation between family physician supply per 10 000 population and inpatient crude death rate, for time period 2.

Parameter	B	s.e.	95% Wald confidence interval		Hypothesis test		
			Lower	Upper	Wald Chi-square	df	Sig.
(Intercept)	3.250	0.3644	2.536	3.964	79.527	1	0.000
FP_time2	−0.024	0.0251	−0.073	0.025	0.932	1	0.334
[Province=EC]	3.129	0.4496	2.248	4.011	48.437	1	0.000
[Province=FS]	2.660	0.4830	1.713	3.606	30.325	1	0.000
[Province=GP]	2.407	0.4788	1.469	3.346	25.275	1	0.000
[Province=KZN]	2.338	0.4075	1.540	3.137	32.930	1	0.000
[Province=LP]	2.352	0.4860	1.400	3.305	23.422	1	0.000
[Province=MP]	2.611	0.5591	1.515	3.706	21.805	1	0.000
[Province=NC]	1.574	0.4971	0.600	2.549	10.027	1	0.002
[Province=NW]	3.550	0.5168	2.537	4.562	47.179	1	0.000
[Province=WC]	0^a^						
(Scale)	0.625^b^	0.1226	0.426	0.918			

Dependent variable: Crude death rate_time 2 Model: (Intercept), FP_time2, Province.

a , Set to zero because this parameter is redundant.

b , Maximum likelihood estimate.

Sig., significance level; df, degrees of freedom; s.e., standard error; B, regression; EC, Eastern Cape; FS, Free State; GP, Gauteng Province; KZN, KwaZulu-Natal; LP, Limpopo Province; MP, Mpumalanga; NC, Northern Cape; NW, North West; WC, Western Cape.

**TABLE 6:** Generalised linear model (regression analysis) to control for the effect of province on the correlation between changes in family physician supply per 10 000 population and percentage of TB cases with known HIV status.

Parameter	B	s.e.	95% Wald confidence interval		Hypothesis test		
			Lower	Upper	Wald Chi-square	df	Sig.
(Intercept)	15.143	3.8935	7.512	22.774	15.127	1	0.000
[Province=EC]	8.552	5.0515	−1.349	18.453	2.866	1	0.090
[Province=FS]	5.782	5.6366	−5.265	16.830	1.052	1	0.305
[Province=GP]	6.294	5.6398	−4.760	17.348	1.246	1	0.264
[Province=KZN]	7.198	4.6770	−1.968	16.365	2.369	1	0.124
[Province=LP]	6.616	5.6577	−4.473	17.705	1.367	1	0.242
[Province=MP]	5.978	6.4898	−6.741	18.698	0.849	1	0.357
[Province=NC]	1.777	5.9560	−9.896	13.451	0.089	1	0.765
[Province=NW]	10.369	6.0384	−1.466	22.204	2.949	1	0.086
[Province=WC]	0^a^						
FPppop_change	−138.039	63.2795	−262.065	−14.014	4.759	1	0.029
(Scale)	83.979^b^	16.4696	57.179	123.340			

Dependent variable: TB known HIV status_difference Model: (Intercept), Province, FPppop_change.

a , Set to zero because this parameter is redundant.

b , Maximum likelihood estimate.

Sig., significance level; df, degrees of freedom; s.e., standard error; B, regression; EC, Eastern Cape; FS, Free State; GP, Gauteng Province; KZN, KwaZulu-Natal; LP, Limpopo Province; MP, Mpumalanga; NC, Northern Cape; NW, North West; WC, Western Cape.

## Discussion

### Key findings

Five years after the introduction of family physicians this study showed no demonstrable correlation between family physician supply and improved health indicators from the macro-perspective of the district. The lack of a measurable impact at the level of the district is most likely because of the very low supply and deployment of family physicians in the public sector, which makes their impact undetectable.

### Discussion of key findings

The family physician supply in the international literature (supply ranging between 4.3 and 12.0 per 10 000 population in countries such as the USA, UK, Canada and Korea) was at least 100 times more than the 0.03 per 10 000 reported here. Our definition of family physician supply, however, differed from the definitions of primary care physician supply in these references, as the international literature generally included all clinical primary care physicians (usually with postgraduate training in specialities such as paediatrics and internal medicine). These international studies were also conducted in less socio-economically deprived settings where postgraduate training of primary care physicians was well established. It may be more appropriate to compare our family physician supply to that of other BRICS countries (Brazil, Russia, India, China and South Africa): the total family physician supply in South Africa (private and public sector, all levels of health care) was 0.1 per 10 000 in 2015, compared to 0.2 per 10 000 in Brazil and 1.2 per 10 000 in China.^39^ The total South African supply of family physician per 10 000 needs to double in order to meet at least Brazil’s supply. South Africa’s NDoH echoes this by identifying a shortfall of 888 family physicians in their 2011 HR policy document.^30^

While some correlations demonstrate a possible trend, the size of these correlations did not exceed 0.5 in either direction. The initial significant correlations disappeared after controlling for the available confounders, especially the provincial covariate. This large degree of heterogeneity between the provinces makes it difficult to assess for an effect of the family physician supply per 10 000 population at a country level.

### Strengths and limitations

Our study was limited by our definition of primary care physician supply, by excluding primary care doctors who were not registered as family physicians with the Health Professions Council of South Africa. A further limitation is the exclusion of private sector family physicians who may have an indirect effect on DHS performance, as they are seeing uninsured patients for out-of-pocket consultations. Some private sector family physicians may be contracted into public sector primary care facilities in the NHI pilot districts since 2013.

The study was also limited by the set of DHB variables that were determined by the NDoH and were not specifically intended to measure the impact of the family physician. The DHB data are based on routinely collected data which may lack the rigour required for research, although HST applies statistical methods to clean and improve data quality. Data quality issues of source data were described in the DHB.^35^ Furthermore, our analysis was limited by the availability of data for all indicators in both time periods, as an analysis over time is more sensitive to the effect of family physicians as opposed to a cross-sectional analysis.

### Implications or recommendations

While this study from a broad macro-level district perspective did not demonstrate an impact of the family physicians on the DHS performance, other studies to be published elsewhere will present additional data from the facility and individual levels. These studies at a meso-level and a micro-level are more likely to demonstrate an impact as they evaluate the family physicians closer to their circle of control and influence. The correlation analysis should be repeated in 5 years, when the family physician supply is greater. It is also recommended that this correlation analysis includes a comparison with a broader definition of primary care doctor supply (all primary care doctors working in the DHS).

## Conclusion

It is still too early to demonstrate the impact of an increase in supply of family physicians at the district level on key health system performance indicators, key clinical processes and key health outcomes. Studies which evaluate impact closer to the family physician’s circle of control may be better positioned to demonstrate a measurable impact in the short term. A repeat correlation analysis is recommended in 5 years to allow for time (duration of effect) and training output (size of supply). Opportunities to deploy more family physicians within the DHS should be explored and supported.
